# PB.34: Results by letter for low-risk breast biopsies: an audit of current practice at Nottingham Breast Institute

**DOI:** 10.1186/bcr3534

**Published:** 2013-11-08

**Authors:** WK Al-Obaydi, EJ Cornford, SL Tennant

**Affiliations:** 1 Nottingham Breast Institute, City Hospital, Nottingham, UK

## Introduction

Patients undergoing breast biopsy are usually asked to return to the results clinic, to enable face-to-face counselling and support. However, a significant proportion of biopsies yield benign or normal results. Since 2011 we have offered the choice of results by letter to selected patients undergoing breast biopsy. This is at the discretion of the assessing radiologist or surgeon, and includes radiologically guided biopsy of benign/probably benign lesions and clinical biopsies where the imaging is normal. Our aim was to identify our accuracy in assessing a patient as suitable for results by letter.

## Methods

Retrospective review of 100 consecutive patients entering this pathway between January and May 2013. Patients were identified from MDT lists, and hospital RIS and pathology systems were interrogated.

## Results

One hundred females, age range 20 to 79 years (mean 42), were included. Eighty-nine presented symptomatically, 11 were recalled from screening. Ninety-two core biopsies and eight fine needle aspirates (FNAs) were performed under ultrasound guidance (91 patients), stereotactically (two patients) or freehand (seven patients with normal imaging). Cytology/histology was normal or benign in 98 patients. Two patients were recalled following FNA results of C4 and C5 and were subsequently diagnosed with DCIS and invasive cancer, respectively.

## Conclusion

Our current practice of identifying low-risk patients is 98% accurate. Sending selected results by letter can obviate the need for a results clinic appointment, with benefits to both patients and staff. However, a robust method of tracking these patients is vital, as well as thorough MDT discussion.

